# FCTP-WSRC: Protein–Protein Interactions Prediction *via* Weighted Sparse Representation Based Classification

**DOI:** 10.3389/fgene.2020.00018

**Published:** 2020-02-04

**Authors:** Meng Kong, Yusen Zhang, Da Xu, Wei Chen, Matthias Dehmer

**Affiliations:** ^1^ School of Mathematics and Statistics, Shandong University at Weihai, Weihai, China; ^2^ University of Applied Sciences Upper Austria, School of Management, Steyr, Austria; ^3^ College of Artificial Intellegience, Nankai University, Tianjin, China; ^4^ Department of Biomedical Computer Science and Mechantronics, UMIT Hall, Tyrol, Austria

**Keywords:** protein–protein interactions, principal component analysis, sparse representation, prediction, crossover network

## Abstract

The task of predicting protein–protein interactions (PPIs) has been essential in the context of understanding biological processes. This paper proposes a novel computational model namely FCTP-WSRC to predict PPIs effectively. Initially, combinations of the F-vector, composition (C) and transition (T) are used to map each protein sequence onto numeric feature vectors. Afterwards, an effective feature extraction method PCA (principal component analysis) is employed to reconstruct the most discriminative feature subspaces, which is subsequently used as input in weighted sparse representation based classification (WSRC) for prediction. The FCTP-WSRC model achieves accuracies of 96.67%, 99.82%, and 98.09% for *H. pylori*, *Human* and *Yeast* datasets respectively. Furthermore, the FCTP-WSRC model performs well when predicting three significant PPIs networks: the single-core network (CD9), the multiple-core network (Ras-Raf-Mek-Erk-Elk-Srf pathway), and the cross-connection network (Wnt-related Network). Consequently, the promising results show that the proposed method can be a powerful tool for PPIs prediction with excellent performance and less time.

## Introduction

Investigating protein–protein interactions (PPIs) relate to examine the correlation between proteins involved in various aspects of life processes such as signal transduction, gene expression regulation, energy metabolism, and cell cycle regulation. The traditional way of studying individual proteins has failed to meet the requirements of the post-genome era because the performance of proteins is diverse and dynamic when performing physiological functions. Therefore, proteins should be studied at the global, network, and dynamic levels. Only by studying the sum of all proteins can we support the understanding of life's behavioral processes, disease prevention, and development of new drugs (Long et al., 2019). In recent years, some researchers predict PPIs by biological methods such as yeast two-hybrid screening (Ito et al., 2001; Pazos and Valencia, 2002) and affinity purification (Gavin et al., 2002). However, the results obtained by wet-lab experiments usually contain a large amount of false positive and false negative data, and these methods are time consuming and costly. These limitations motivate the development of effective machine learning methods to predict large-scale PPIs.

Up to now, D.S. Huang et al. predicts PPIs utilizing different information sources such as tertiary structure of proteins, phylogenetic profiles, and protein domains (De-Shuang and Chun-Hou, 2006; De-Shuang and Ji-Xiang, 2008). However, these computational methods require prior knowledge of the target protein (An et al., 2016). In recent years, protein sequence-based methods (Yu et al., 2017) are becoming the most widely applied technique for predicting PPIs due to the availability of protein sequence data. Liu et al. (2012) designs a sequence analysis method to represent protein sequences based on hypergeometric series using the q-Wiener index (Xu et al., 2017). X. Li et al. employs a global encoding approach (GE) to describe global information of amino sequence (Li et al., 2009).

Since the effectiveness of machine learning algorithms has been continuously verified in recent years, the use of machine learning methods for predicting PPIs has become a new research area. Yanzhi et al. proposes a support vector machine (SVM) prediction method based on auto covariance (AC) (Wold et al., 1993; Yanzhi et al., 2008) Davies et al. designs a model based on k-nearest neighbor (KNN) with local descriptor (LD) (Juan et al., 2007; Davies et al., 2008; Tong and Tammi, 2008; Lei et al., 2010). Juwen et al. using SVM with conjoint triad method predicting PPIs (Juwen et al., 2007). In addition, algorithms that use machine learning include: random forest (RF) with multi scale continuous and discontinuous local descriptor (MCD) (You et al., 2014), deep neural networks (DNNs) with pseudo amino acid physicochemical property descriptors(APAAC) (Kuo-Chen, 2005; Du et al., 2017) and so forth. These methods to perform PPIs prediction use solely amino acid sequence data. In addition, different representation methods can extract distinct characteristic information of protein sequences, and it is known that the feature information extracted by these representation methods can be complementary. Thus, for PPIs prediction, we advocate combining multiple descriptors, which can capture more information than a single descriptor (Deng et al., 2015). EnsDNN is a multi-descriptor combining method based on deep neural network (Xenarios et al., 2002). These descriptors such as auto-covariance descriptor (AC), local descriptor (LD) and multi-scale continuous and discontinuous local descriptor (MCD). It achieved a high accuracy of 95.25% on the *Saccharomyces cerevisiae* dataset. Despite this, there is still room to improve the accuracy and efficiency.

Previous works have pointed out that using feature selection or feature extraction before conduction the classification tasks can improve the classification accuracy (Zhang et al., 2012). The software EFS (Ensemble Feature Selection) makes use of multiple feature selection methods and combines their normalized outputs to a quantitative ensemble importance. Currently, eight different feature selection methods have been integrated in EFS, which can be used separately or combined in an ensemble (Neumann et al., 2017). What's more, several evolutionary based methods are proposed for dimensionality reduction (Chuang et al., 2016). A multi-objective differential evolution method (called MODEMDR) was proposed to merge the various contingency table measures based on MDR to detect significant gene-gene interactions (Yang et al., 2017). In this paper, principal component analysis (PCA) is utilized to do the feature extraction which projects the original feature space into a new space. The effectiveness of the proposed FCTP-WSRC is examined in terms of classification accuracy on the PPI dataset.

The main contribution of this paper is to develop a new computational tool called FCTP-WSRC to predict PPIs efficiently. More precisely: (1) Combinations of the F-vector, composition (C) and transition (T) are used to map each protein sequence on numeric feature vectors. (2) An effective feature extraction method PCA (principal component analysis) is employed to reconstruct the most discriminative feature subspaces, which is subsequently used as input in weighted sparse representation based classification (WSRC) for prediction. We obtain a unique 60-dimensional feature vector of each protein pair. (3) The FCTP-WSRC model can predict newly discovered protein-protein interactions with unknown biological functions using only protein sequence information.

## Methodology

### Reduced Sequence and F-Vector

In this paper, a computational model based on multivariate mutual information is designed to represent the protein sequence and obtain the feature vector. The model describes the protein sequence as a fixed length feature vector containing key information, which can be used as an effective input for machine learning algorithm. Therefore, the design of the F vector, the composition and transition (CT) descriptors is combined to map each protein sequence to a digital feature vector. F-vector of protein sequence is constructed in the following manner.

First, we generate reduced amino acid sequences according to their physicochemical properties such as hydrophobicity and polarity. When studying Shannon entropy of residue properties, instead of treating the amino acids as distinct symbols in the entropy calculation, six groups have proposed partitioning the amino acids into stereo chemically defined sets, and then computing the entropy of the column with respect to these sets. According to Capra JA et al. (Capra and Singh, 2007), we classify residues into six different classes. The six classes of amino acids are: aliphatic (AVLIMC), aromatic (FWYH), polar (STNQ), positive (KR), negative (DE), and special (reflecting their special conformational properties) (GP) (Mirny and Shakhnovich, 1999), as depicted in **Table 1**.

**Table 1:** Amino acid classification.

Descriptor	Property	Classification
A1	Aliphatic amino acid	A,V,L,I,M,C
A2	Aromatic amino acid	F,W,Y,H
A3	Polar amino acid	S,T,N,Q
A4	Positive amino acid	K,R
A5	Negative amino acid	D,E
A6	Special conformations	G,P

The plane rectangular coordinate system has four quadrants. Dividing 20 amino acids into four groups can use the formula (1) to map the protein sequence to the unit circle. However, 20 amino acids are divided into six classes. Thus, we recombine six types of amino acids. Three classes of amino acids are selected from the six classes of amino acids as one group and the remaining three classes are unchanged. In this way, we can get four groups of amino acids, and there are a total of 20 combination patterns. It is found through experiments that the 20 patterns will cause too many features and affect the operation efficiency. Selecting the top 10 combination patterns got good results.

Then, we use a binary space (*V*, *F*) to describe amino acid sequences. Here, *V* is the feature space of the sequence information, and each amino acid combined pattern *v_i_* represents a sort of quad type; *F* is the feature vector corresponding to *V*. The size of *V* should be 10; thus, *I =* 1,2,…, 10. We describe ten amino acid combined patterns by the letters B, J, O and U in **Table 2**. The detailed definition and description for (*V*, *F*) are illustrated by the Equations (1)-(4). Clearly, each protein has a corresponding *F* vector.

(1) $$S_{q}(v_{i})\to \{\begin{array}{ll} \left(\cos(\frac{\pi }{2}\frac{B_{j}}{B_{n}+1}),\sin(\frac{\pi }{2}\frac{B_{j}}{B_{n}+1})\right) & ifS_{q}=B\\ \left(\cos(\frac{\pi }{2}+\frac{\pi }{2}\frac{J_{j}}{J_{n}+1}),\sin(\frac{\pi }{2}+\frac{\pi }{2}\frac{J_{j}}{J_{n}+1})\right) & ifS_{q}=J\\ \left(\cos(\pi +\frac{\pi }{2}\frac{O_{j}}{O_{n}+1}),\sin(\pi +\frac{\pi }{2}\frac{O_{j}}{O_{n}+1})\right) & ifS_{q}=O\\ \left(\cos(\frac{3\pi }{2}+\frac{\pi }{2}\frac{U_{j}}{U_{n}+1}),\sin(\frac{3\pi }{2}+\frac{\pi }{2}\frac{U_{j}}{U_{n}+1})\right) & ifS_{q}=U \end{array}$$

**Table 2:** Ten amino acid combined patterns described by the letters B, J, O, and U.

	*B*	*J*	*O*	*U*
*v*_1_	{*A*_1_, *A*_2_, *A*_3_}	*A*_4_	*A*_5_	*A*_6_
*v*_2_	{*A*_1_, *A*_2_, *A*_4_}	*A*_3_	*A*_5_	*A*_6_
*v*_3_	{*A*_1_, *A*_2_, *A*_5_}	*A*_3_	*A*_4_	*A*_6_
*v*_4_	{*A*_1_, *A*_2_, *A*_6_}	*A*_3_	*A*_4_	*A*_5_
*v*_5_	{*A*_1_, *A*_3_, *A*_4_}	*A*_2_	*A*_5_	*A*_6_
*v*_6_	{*A*_1_, *A*_3_, *A*_5_}	*A*_2_	*A*_4_	*A*_6_
*v*_7_	{*A*_1_, *A*_3_, *A*_6_}	*A*_2_	*A*_4_	*A*_5_
*v*_8_	{*A*_1_, *A*_4_, *A*_5_}	*A*_2_	*A*_3_	*A*_6_
*v*_9_	{*A*_1_, *A*_4_, *A*_6_}	*A*_2_	*A*_3_	*A*_5_
*v*_10_	{*A*_1_, *A*_5_, *A*_6_}	*A*_2_	*A*_3_	*A*_4_

We suppose each reduced sequence *S*=*S*_1_ *S*_2_ *S*_3_⋯*S*_*n*_, *S*_*q*_∈{*B*, *J*,*O*,*U*}, and *q* = 1, 2,…, *n*. *B_n_* is the number of *B* in the sequence *S* by using the pattern *v_i_*. *B_j_* is the number of *B* in the first *j* characters when *S_j__=_ B*. According to Equation (1), we introduce Equation (2):

(2) $$S(v_{i})\to \{\begin{array}{l} M_{x}=\frac{1}{n}\sum_{q=1}^{n}x_{q}\\ M_{y}=\frac{1}{n}\sum_{q=1}^{n}y_{q}\\ V_{x}=\sqrt{\frac{1}{n-1}\sum_{q=1}^{n}{\left(x_{q}-M_{x}\right)}^{2}}\\ V_{y}=\sqrt{\frac{1}{n-1}\sum_{q=1}^{n}{\left(y_{q}-M_{y}\right)}^{2}} \end{array}$$

Here *x_q_* and *y_q_* (*q =* 1,2,⋯, *n*) are derived from Equation (1).

**Figure 1:**
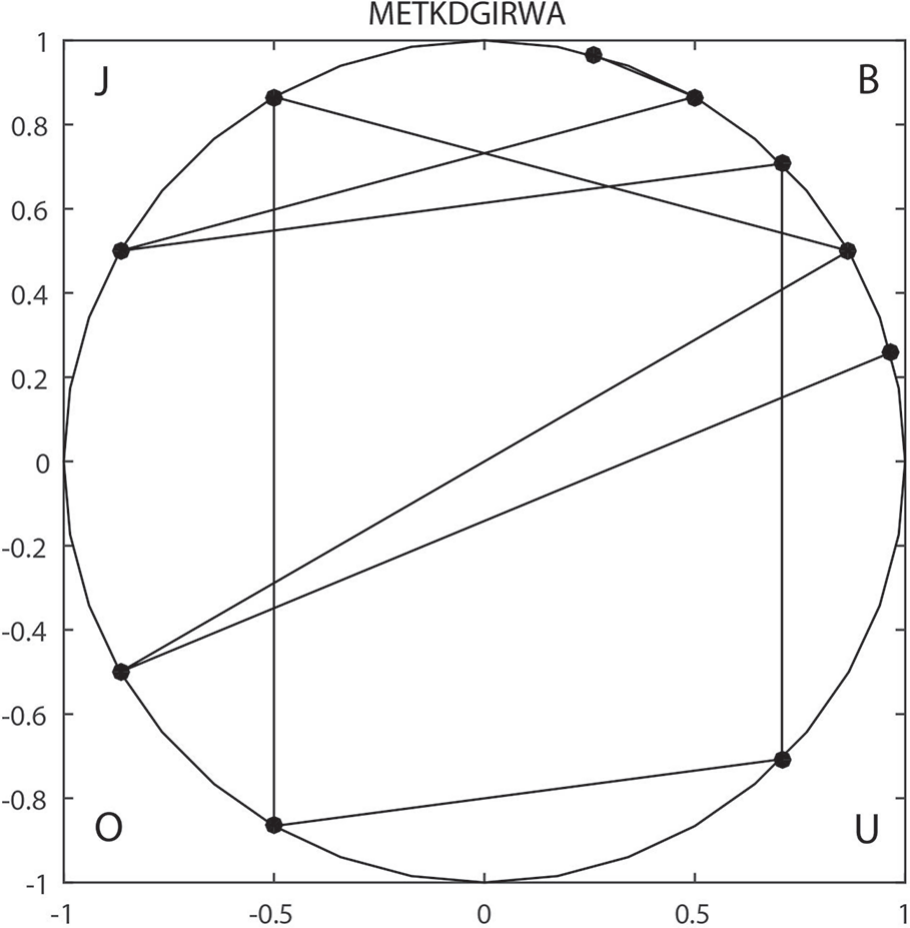
2-D Unit circle mapping representation of “METKDGIRWA” under pattern.

For example, sequence *METKDGIRWA* can be expressed as *BOBJOUBJBB* based on *v*_1_, so it is mapped to the unit circle as shown in **Figure 1**. The reduced sequence corresponds to a one-to-one curve in the unit circle. So, the invariant of the curve can be used as the characteristic value of the sequence. Finally, the F-vector can be expressed by:

(3) $$F=(F(v_{i}),F(v_{2}),\cdots ,F(v_{10}))$$

The vector *F*(*v_i_*) is as follows:

(4) $$F(v_{i})=(M_{x},M_{y},V_{x},V_{y}),i=1,2,\cdots ,10$$

Thus, a 40-dimensional vector is obtained to characterize each amino acid sequence.

### The Composition and Transition of Protein Sequence (CT)

In this section, we put forward a new description approach using binary coding sequences. First of all, the amino acid sequence is mapped to a sparse matrix. Then the composition (C) and transition (T) of characteristic sequence are extracted from the obtained sparse matrix. The protein sequence is scanned from left to right by the step of one amino acid at a time. Suppose a protein sequence with *n* amino acid residues is given: *S*=*S*_1_ *S*_2_ *S*_3_⋯*S*_*n*_;*D =* {*A*,*R*,*N*,*D*,*C*,*E*,*Q*,*G*,*H*,*I*,*L*,*K*,*M*,*F*,*P*,*S*,*T*,*W*,*Y*,*V*}. Now we derive the matrix *A* of this sequence:

$$A={\left(\begin{array}{lllllll} \begin{array}{l} A\\ R\\ N \end{array} & \begin{array}{l} S_{1}\\ a_{11}\\ a_{21}\\ a_{31} \end{array} & \begin{array}{l} S_{2}\\ a_{12}\\ a_{22}\\ a_{32} \end{array} & \begin{array}{l} S_{3}\\ a_{13}\\ a_{23}\\ a_{33} \end{array} & \begin{array}{l} \cdots \\ \cdots \\ \cdots \\ \cdots \end{array} & \begin{array}{l} S_{n-1}\\ a_{1,n-1}\\ a_{2,n-1}\\ a_{3,n-1} \end{array} & \begin{array}{l} S_{n}\\ a_{1,n}\\ a_{2,n}\\ a_{3,n} \end{array}\\ \vdots & \vdots & \vdots & \vdots & \ddots & \vdots & \vdots \\ \begin{array}{l} Y\\ V \end{array} & \begin{array}{l} a_{19,1}\\ a_{20,1} \end{array} & \begin{array}{l} a_{19,2}\\ a_{20,2} \end{array} & \begin{array}{l} a_{19,3}\\ a_{20,3} \end{array} & \begin{array}{l} \cdots \\ \cdots \end{array} & \begin{array}{l} a_{19,n-1}\\ a_{20,n-1} \end{array} & \begin{array}{l} a_{19,n}\\ a_{20,n} \end{array} \end{array}\right)}_{20*n}$$

(5) $$a_{i,j}=\;\{\begin{array}{ll} 1, & ifD(i)=S(j)\\ 0, & others \end{array}$$

where *D*(*i*) is the *i*-th kind of amino acid in the arranged letter sequence *D*.

**Figure 2:**
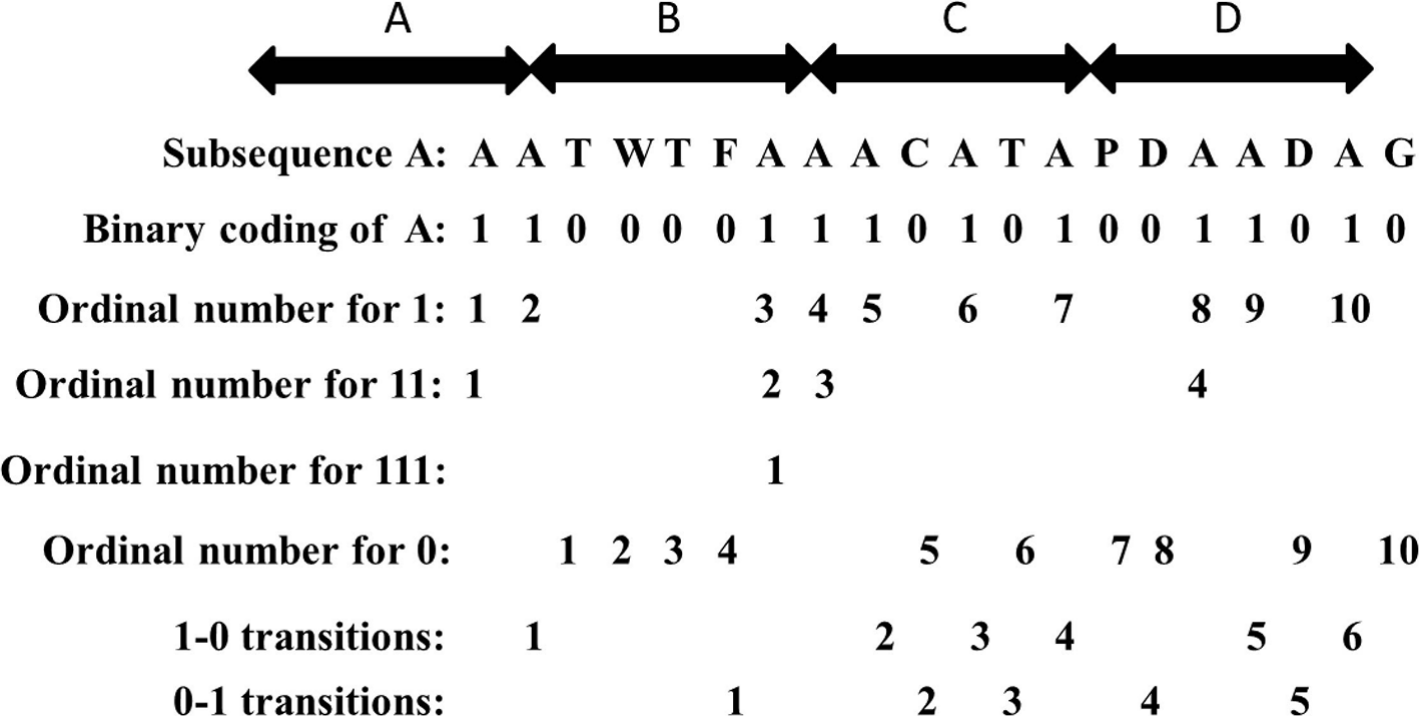
The composition and transition of subsequence “AATWTFAAACATAPDAADAG” with respect to amino acid A.

For each row vector of matrix A with length *n*, we divide the sequence into *L* sub-vectors. For each characteristic sub-vector, the composition (C) consists of four parts: frequency of “0”, frequency of “1”, frequency of “11” and frequency of “111”, respectively. The descriptor (T) is the frequency of “0” followed by “1” or “1” followed by “0”. An example regarding the composition (C) of the sub-vector with respect to amino acid A is shown in the **Figure 2**. The subsequence “AATWTFAAACATAPDAADAG” with respect to amino acid A is replaced by “11000011101010011010”. We see that there exists ten “1”, ten “0”, four “11”, and one “111”. The composition for these four parts is 10×100%/(10 + 10) = 50%, 10×100%/(10 + 10) = 50%, 4×100%/19 = 21.05%, and 1 × 100%/18 = 5.56%. The transition for “1-0” and “0-1” is (6 + 5)×100%/19 = 57.89%. Thus, a protein sequence is transformed into a 4×20×5 = 400 dimensional vector with *L =* 4 and 20 amino acids.

### Reconstructing Feature Vectors

So far, we combine the descriptor F-vector (40 dimension) and descriptor CT (400 dimension) for a protein sequence into a 440-dimensional vector. However, if this vector is used as input of the classifier directly, the efficiency is likely to be low. Therefore, in this section we discuss how to reconstruct new feature vectors using principal component analysis (PCA). Principal component analysis (PCA) is a widely used dimensional compression technique. The main idea of PCA is to sequentially find a set of mutually orthogonal coordinate axes from the original space, which is closely related to the data itself. When 30 dimensional features are selected, the contribution rate of features can reach more than 90%. It can not only ensure the accuracy, but also improve the calculation efficiency. Therefore, we use PCA to reduce 440 dimension vector to 30 dimension. We connect the feature vectors of two proteins (*V_A_* and *V_B_*) to describe their interaction information (*V_AB_*):

(6) $$\left\{V_{AB}\}=\{V_{A}\}⊕\{V_{B}\right\}$$

Thus, a pair of proteins can be expressed by a 60 dimensional vector.

### Weighted Sparse Representation Based Classification (WSRC)

In recent years, inspired by the theory of compressed sensing, Wright et al. (2009) proposed a sparse representation based classification (SRC). The algorithm has been proven useful and reliable for many applications. Later, Fan et al. (2015) proposed a weighted sparse representation based classification (WSRC), which introduced sample weights into training samples and enhanced the robustness of classification. Usually the representation result of WSRC is sparser than that of SRC, so better recognition results can be obtained. Here we give a brief introduction towards WSRC.

Suppose that training samples are classified into *C* classes. Let *X* = [*X*_1_, *X*_2_,…, *X*_c_] ∈ *R^d^* ^x^ *^n^*, where *X_i_* ∈ *R^d^* ^x *n i*^ is the *n_i_* training sample of class *i*. Given a test sample *y* ∈ *R^d^*: *y* = *X*α, where α = [α_1_, α_2_,…, α_c_], α*_i_* is the representation coefficient vector associated with the *i*-th class. WSRC keeps data relativity while sparse representation makes coding localized and allows more neighboring samples to express the samples to be tested. The training samples nearer to the test samples should be given smaller weights to make their corresponding coefficients larger. The objective function is:

(7) $$(Weighted\;l^{1}):\min\text{ ||W}\alpha |{|}_{1}$$

subject to

(8) y=Xα

Dealing with occlusion, the Equations (7) and (8) should be extended to the stable *l*\s\do5(1)−*minimization* problem:

(9) $$\hat{\alpha }=\arg\;min\text{ ||}\alpha {\text{||}}_{1}$$

subject to

(10) ∥y−Xα∥≤ ϵ.

**ε** > 0 is the tolerance of reconstruction error. After obtaining the sparsest solution $\hat{\alpha }$, we assign a test sample *y* to the class *i* by the following rule:

(11) $$min_{i}r_{i}(y)=\;∥y-X{\hat{\alpha }}^{i}\|,i=1,2,\ldots ,c.$$

and specifically,

(12) $$diag(W)=[d(y,x_{1}^{1}),\ldots ,d(y,x_{n_{c}}^{c})].$$

*W* is a diagonal matrix used to adjust the weight of training samples to express the test samples and *n_c_* is the sample number of training set in class *c*. WSRC calculates the Gaussian similarities between the test sample and the entire training samples, which are used as the weight of each training sample. The Gaussian similarity between two samples, a1 and a2, could be defined as follows:

(13) $$d\left(a_{1},a_{2}\right)=\exp\;\left(-\frac{∥a_{1}-a_{2}{∥}^{2}}{2\sigma^{2}}\right)$$

where σ means the Gaussian kernel width. In this paper, we take the parameters ϵ = 0.005, σ = 1.5. The WSRC algorithm can be described as follows:

**Algorithm 1:** Weighted sparse representation based classification (WSRC).

**INPUT:**
The matrix of training samples *X*∈*R* ^*d*×*n*^ and a test sample *y*∈*R* ^*d*^.
**OUTPUT:**
The prediction label of *y* as $identify(y)=\arg\underset{i}{\min}r_{i}(y)$.
1: Normalize each column of *X* to have the unit *l*_2_ norm.
2: Calculate the Gaussian similarity between *y* and each sample in *X* and obtain the weight matrix *W*.
3: Solve the stable *l*_1_—minimization problem described in Equation (7).
4: Calculate residual error: $\underset{i}{\min}r_{i}(y)=∥y-X{\hat{\alpha }}^{i}∥,\;i=1,2,...,c.$
5: **return** *y*;

## Dataset

In this paper, *H. pylori*, *Yeast*, and *Human* PPIs datasets are downloaded from the DIP database (Xenarios et al., 2002). Cd-hit (Li et al., 2001) is a tool for protein sequence clustering that clusters sequences based on their similarity. This article uses the cd-hit tool to remove redundant sequences such that the protein interaction dataset has less than 40% homology and builds a non-redundant dataset (Shawn et al., 2005). Thus, the *H. pylori* dataset contains 1,428 pairs of interacting proteins, the *Yeast* dataset contains 5,594 pairs of interacting proteins, and the *Human* dataset contains 3,899 pairs of interacting proteins. The choice of negative samples is crucial. This paper constructs a non-interacting dataset (negative sample) based on the protein interaction dataset (positive sample) that has been obtained (Yanzhi et al., 2008; You et al., 2015). Sequences in non-interacting protein pairs are randomly selected from a positive samples, but several conditions need to be met: (1) Non-interacting sequence pairs cannot appear in the interaction dataset. (2) The number of protein pairs in a non-interacting dataset should be balanced with the interacting dataset. (3) The contribution of each protein sequence in the non-interacting dataset should be as consistent as possible. Through this strategy, 1458 negative samples of *H. pylori*, 5,594 negative samples of *Yeast*, and 4,262 negative samples of *Human* are obtained. Thus, the *H. pylori* dataset has a total of 2,916 pairs of protein sequences, the *Yeast* dataset has a total of 11,188 pairs of protein sequences, and the *Human* dataset has a total of 8,161 pairs of protein sequences. Furthermore, in order to construct a PPIs network model, three significant PPIs network datasets are performed: the single-core network (CD9), the multiple-core network (Ras-Raf-Mek-Erk-Elk-Srf pathway), and the cross-connection network (Wnt-related Network).

## Evaluation of the Prediction Performance

Here, we employ five fold cross validation to evaluate the performance of the FCTP-WSRC model. The entire dataset is divided into five groups randomly, four of which are used as the training samples and the remaining one as the test samples. The average performance on five sets is used as the performance of our method. Several evaluation indicators are used to evaluate the performance of the development methods of this article. Brief descriptions of these metrics are as follows: (1) sensitivity (Sn) is the percentage of correctly identified interacting protein pairs; (2) specificity (Sp) is the percentage of correctly identified non-interacting protein pairs; (3) accuracy (Acc) is the percentage of correctly identified protein pairs; (4) matthew's correlation coefficient (Mcc) is a stricter evaluation standard considering both under and over predictions. Some concepts and terms to explain this parameters are defined as follows (You et al., 2013):

(14) $$\{\begin{array}{l} Sn=\frac{TP}{TP+FN}\\ Sp=\frac{TN}{TN+FP}\\ Acc=\frac{TP+TN}{TP+FP+TN+FN}\\ Mcc=\frac{\left(TP)(TN)-(FP)(FN\right)}{\sqrt{\left[TP+FP][TP+FN][TN+FP][TN+FN\right]}} \end{array}$$

where TP is the number of true positive; FN is the number of false negative; TN is the number of true negative; and FP is the number of false positive. In addition, the ROC curve and the area under an ROC curve (AUC) (Huang et al., 2016a) are employed to evaluate the performance of the FCTP-WSRC approach.

## Discussion

### Prediction Ability

**Figure 3:**
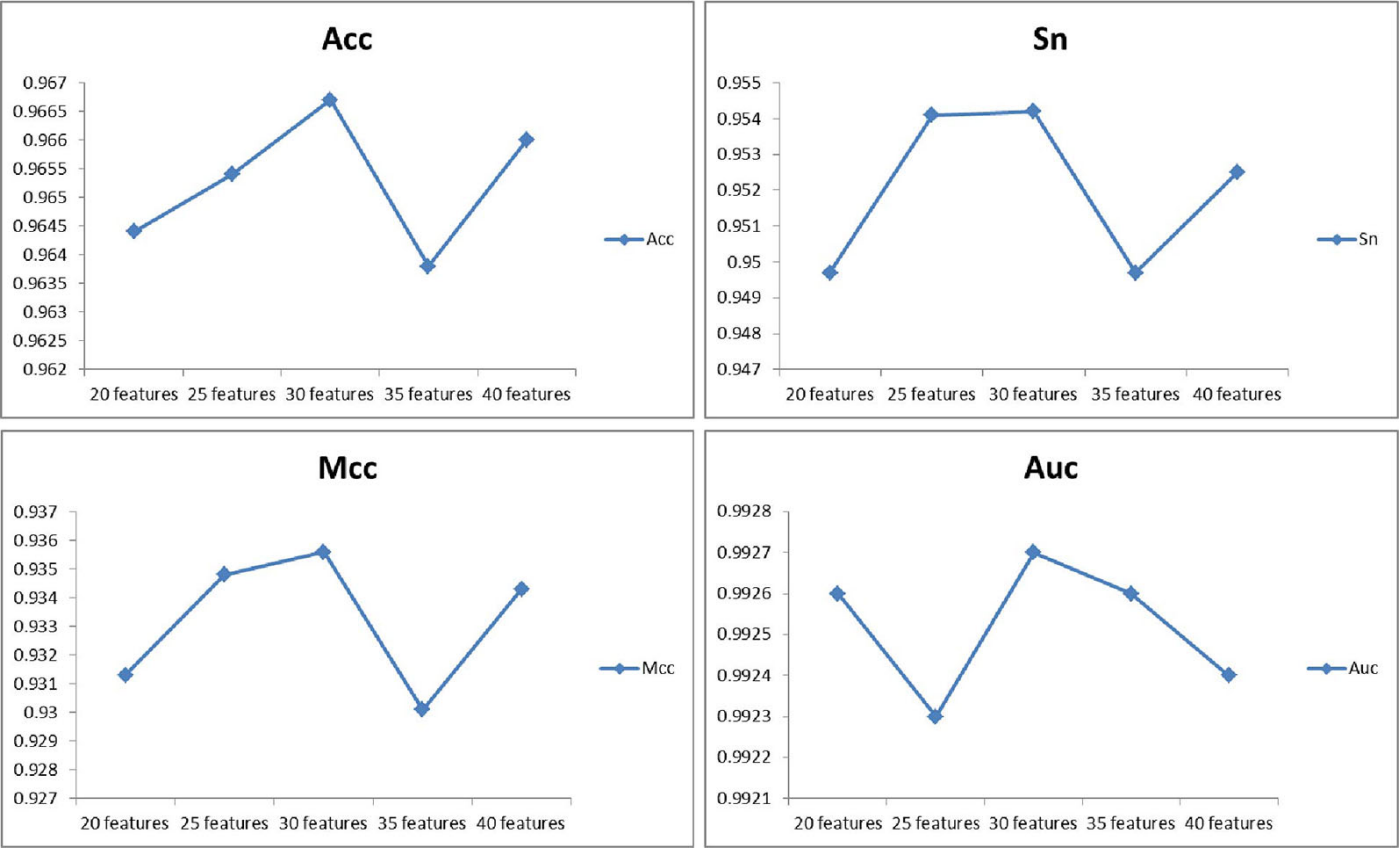
The comparison of the effects of different feature numbers based on principal component analysis (PCA).

For the sake of testing the stability and reliability of the results, we employ a fivefold cross validation for three typical dataset. For the practicality and effectiveness of our proposed method, we conduct ten times five fold cross validations and use the average results as the final experimental results. We obtain the final results of Acc, Sn, Sp, and Mcc of 96.67%, 95.42%, 97.85%, and 93.56% on the *H. pylori* dataset. Moreover, we obtain excellent performance of average accuracy, sensitivity, specificity, and Mcc of 99.82%, 99.88%, 99.77%, 99.63% on the *Human* dataset and 98.09%, 99.45%, 96.82%, 96.25% on the *Yeast* dataset, respectively. What's more, I have compared the feature selection PCA with the current state-of-the-art feature selection methods EFS on the *Human* dataset. The Acc, Sn, Sp and Mcc of EFS are 0.9499, 0.9601, 0.9448, and 0.9045, respectively, which are lower than our method PCA+WSRC. The comparison of the effects of different feature numbers based on PCA is shown in **Figure 3**.

### The Prediction Performance Comparison of FCTP-WSRC With FCTP-SVM

To further verify the effectiveness of the FCTP-WSRC approach, we compare the predictions with the frequently used classifier support vector machine (SVM). The kernel functions commonly used in support vector machines are: linear kernel, polynomial kernel and radial basis kernel function. Linear kernel is mainly used in the case of linear separability. The dataset in this paper has a low feature dimension and is linear inseparability. Compared with the polynomial kernel function, the radial basis kernel function needs to determine fewer parameters, and the more parameters the more complicated the model. Through experiments, we use the LIBSVM (Chang and Lin, 2011) implementation of SVM with the radial basis kernel function:

(15) $$k\;(x,y)=exp(\frac{∥x-\;y{∥}^{2}\|}{2\sigma^{2}})$$

**Figure 4:**
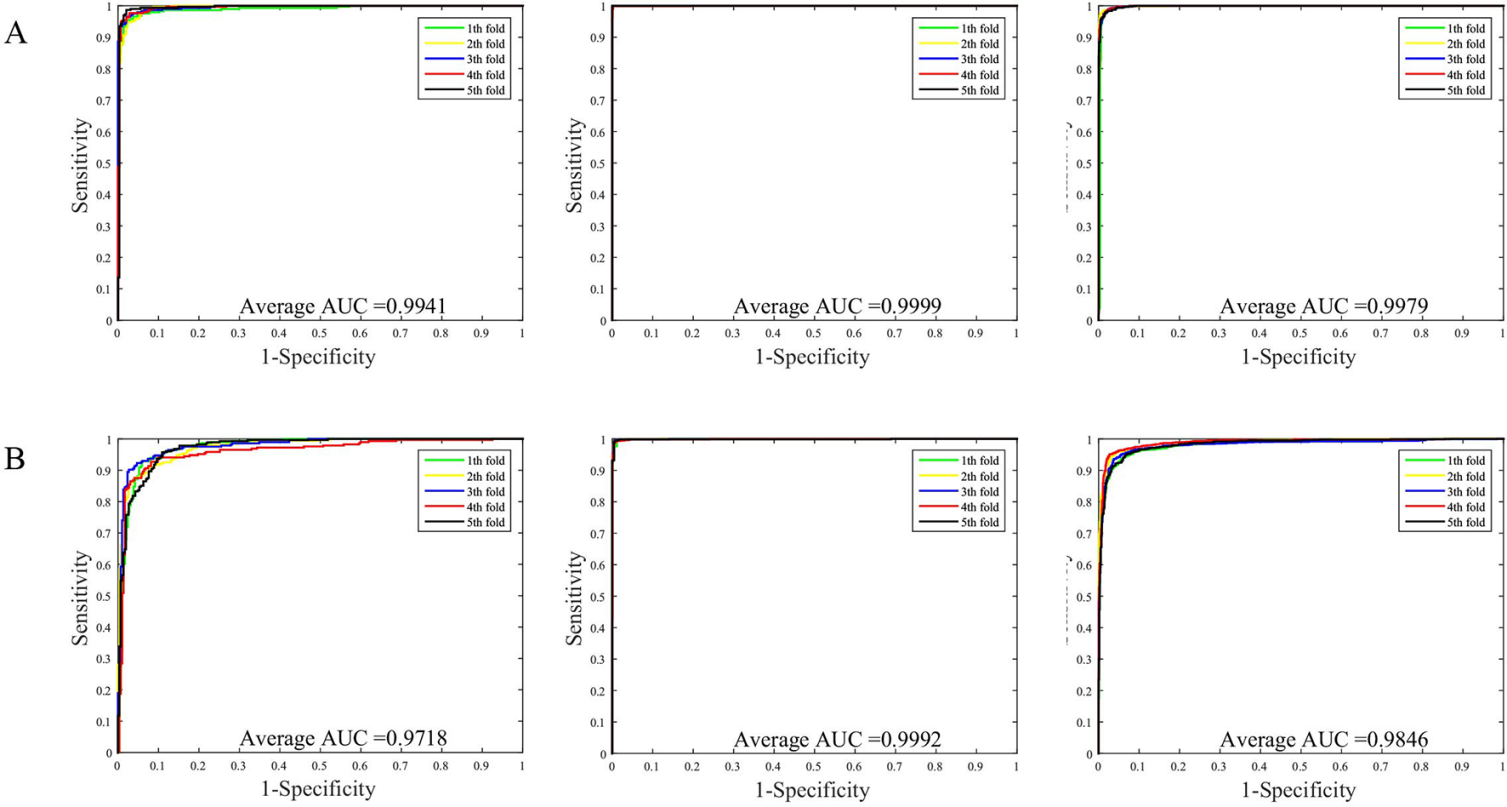
**(A)** ROC curve of FCTP-WSRC on the H. pylori, Human and Yeast datasets. **(B)** ROC curve of FCTP-SVM on the H. pylori, Human and Yeast datasets.

**Figure 5:**
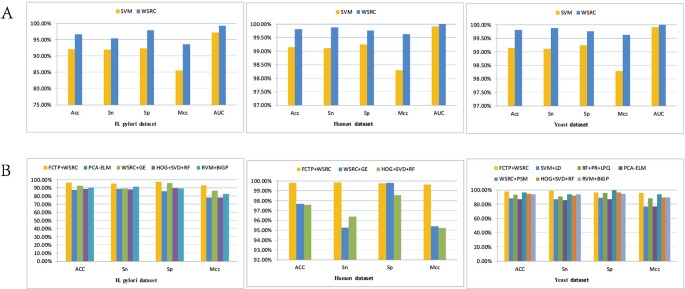
**(A)** Results using FCTP encoding on the H. pylori, Human and Yeast datasets with different classifiers. **(B)** Results using different methods on three datasets.

The prediction results of the SVM and WSRC methods on the H. pylori, Human and Yeast datasets are shown in **Table 3**, and the bar chart is displayed in **Figure 5A**. From these results, we can see that the WSRC classifier is significantly better than the SVM classifier. In addition, the ROC (receive operator characteristic) curve illustrating the performance of different classification methods. The curve presents the sensitivity (the true positive rate) against the specificity (the false positive rate). The ROC curves of FCTP-WSRC on the H. pylori, Human and Yeast datasets are shown in **Figure 4A** and those of FCTP-SVM are shown in **Figure 4B**. Good performance is reflected in curves with stronger bending towards the upper-left corner of the ROC graph, that is, high sensitivity is achieved with a low false positive rate. For all models, the areas under an ROC curves (AUC) are > 97.18%. It can be seen from **Figure 4** that the ROC curves of the WSRC classifier are significantly better than those of the SVM classifier. This clearly prove that the WSRC classifier of the proposed method is an accurate and robust classifier for predicting PPIs. The increased classification performance of the WSRC classifier compared with the SVM classifier can be explained by two reasons: (1) the obvious advantage of WSRC is that it does not need to select and compute kernel functions. (2) Protein sequence data expressed by FCTP method is very sparse, so it is suitable for PPIs prediction by sparse representation classifier.

**Table 3:** The prediction performance comparison of FCTP-WSRC with FCTP-SVM.

Dataset	Classification model	Acc	Sn	Sp	Mcc	AUC
H. pylori dataset	SVM	0.9215	0.9191	0.9235	0.8552	0.9718
	WSRC	**0.9667**	**0.9542**	**0.9785**	**0.9356**	**0.9927**
Human dataset	SVM	0.9914	0.9911	0.9925	0.9830	0.9992
	WSRC	**0.9982**	**0.9988**	**0.9977**	**0.9963**	**1**
Yeast dataset	SVM	0.9482	0.9560	0.9411	0.9019	0.9846
	WSRC	**0.9809**	**0.9945**	**0.9682**	**0.9625**	**0.9986**

Bolded texts are used to emphasize the results of the method designed in this article.

### Comparison With Other Methods

**Tables 4**–**6** compare the prediction performance by the proposed method (FCTP-WSRC) and some outstanding works on the *H. pylori*, *Yeast* and *Human* dataset. **Table 4** describes the average accuracies of other seven methods including HKNN (Nanni, 2005), Signature products (Shawn et al., 2005), Ensemble of HKNN (Nanni and Lumini, 2006), PCA+ELM (You et al., 2013), WSRC+GE (Nanni and Lumini, 2006), HOG+SVD+RF (Ding et al., 2016), and RVM+BiGP (An et al., 2016). **Table 5** describes the average accuracies of other seven methods including LDA+RF (Xiao-Yong et al., 2010), LDA+RoF (Xiao-Yong et al., 2010), AC+RF (Xiao-Yong et al., 2010), AC+RoF [41), WSRC+GE (Huang et al., 2016a), and HOG+SVD+RF (Ding et al., 2016). **Table 6** describes the average accuracies of other seven methods including AutoCC (Yanzhi et al., 2008), SVM+LD (Guo et al., 2015), RF+PR+LPQ (Wong et al., 2015), PCA+ELM (You et al., 2013), WSRC+PSM (Huang et al., 2016b), HOG+SVD+RF (Ding et al., 2016), and RVM+BiGP (An et al., 2016). These results using distinct methods on three datasets are intuitively shown by **Figure 5B**. All the results prove that our method improves predictions by using fixed-length feature vectors.

**Table 4:** Comparing the prediction performance by the proposed method (FCTP-WSRC) and some state-of-art works on the *H. pylori* dataset.

Model	ACC	Sn	Sp	Mcc
Our method	**0.9667**	**0.9542**	**0.9785**	**0.9356**
HKNN	0.8400	0.8600	0.8400	N/A
Signature products	0.8340	0.7990	0.8570	N/A
Ensemble of HKNN	0.8660	0.8670	0.8500	N/A
PCA+ELM	0.8750	0.8895	0.8615	0.7813
WSRC+GE	0.9283	0.8932	0.9613	0.8643
HOG+SVD+RF	0.8906	0.8815	0.8979	0.7815
RVM+BiGP	0.9057	0.9188	0.8955	0.8291

Here, N/A means not available. Bolded texts are used to emphasize the results of the method designed in this article.

**Table 5:** Comparing the prediction performance by the proposed method (FCTP-WSRC) and some state-of-art works on the *Human* dataset.

Model	ACC	Sn	Sp	Mcc
Our method	**0.9982**	**0.9988**	**0.9977**	**0.9963**
LDA+RF	0.9640	0.9420	N/A	0.9280
LDA+RoF	0.9570	0.9760	N/A	0.9180
AC+RF	0.9550	0.9400	N/A	0.9140
AC+RoF	0.9510	0.9330	N/A	0.9100
WSRC+GE	0.9766	0.9528	0.9981	0.9541
HOG+SVD+RF	0.9760	0.9637	0.9859	0.9521

N / A means that the result of this indicator is not queried.

**Table 6:** Comparing the prediction performance by the proposed method (FCTP-WSRC) and some state-of-art works on the *Yeast* dataset.

Model	ACC	Sn	Sp	Mcc
Our method	**0.9809**	**0.9945**	**0.9682**	**0.9625**
AutoCC	0.8933	0.8993	0.8887	N/A
SVM+LD	0.8856	0.8737	0.8950	0.7715
RF+PR+LPQ	0.9392	0.9110	0.9645	0.8856
PCA+ELM	0.8700	0.8615	0.8759	0.7736
WSRC+PSM	0.9709	0.9433	1	0.9433
HOG+SVD+RF	0.9483	0.9240	0.9710	0.8977
RVM+BiGP	0.9457	0.9427	0.9486	0.8974

N / A means that the result of this indicator is not queried.

### Network Prediction

**Figure 6:**
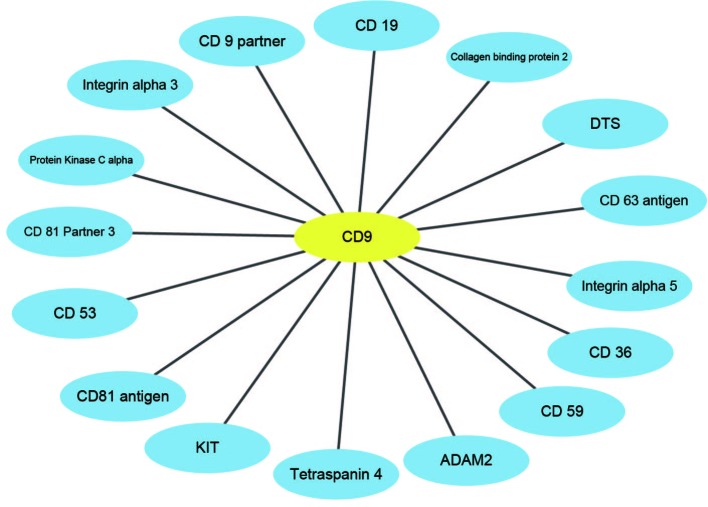
The prediction results of single-core network of CD9.

**Figure 7:**
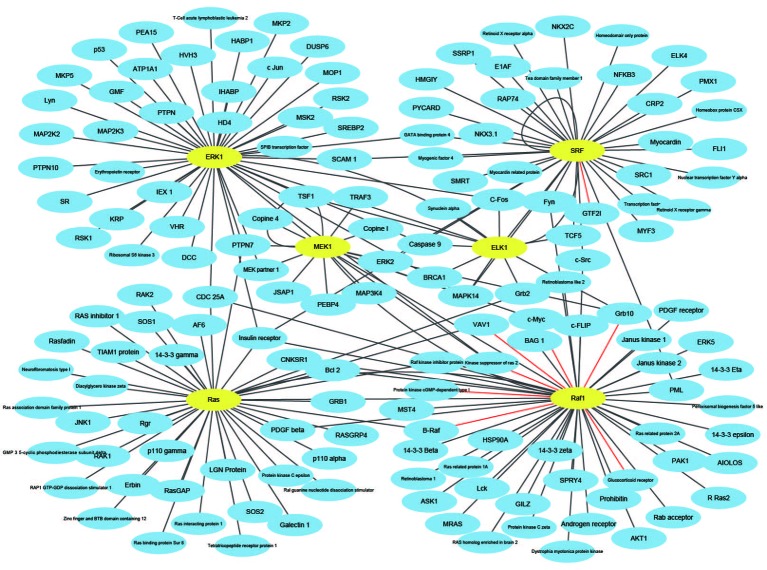
The prediction results of multi-core network of Ras-Raf-Mek-Erk-Elk-Srf pathway.

**Figure 8:**
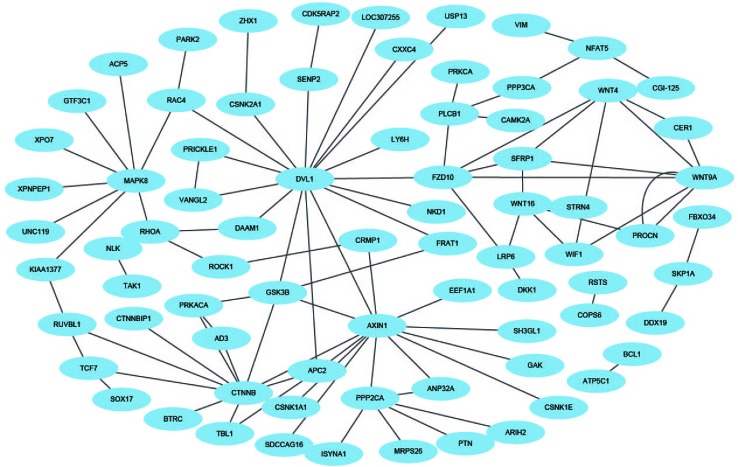
The prediction results of cross-connection network of Wnt-related pathway.

An effective application of a good PPIs prediction method should have a good ability to predict PPI networks. Up to now, many machine learning approaches have been applied to predict PPIs networks. Despite this, there is still room to improve the accuracy and stability. Therefore, we have extended the prediction method of PPI networks consisting of PPI pairs: the single-core network (CD9), the multiple-core network (Ras-Raf-Mek-Erk-Elk-Srf pathway), and the cross-connection network (Wnt-related Network). The prediction results and the networks are shown in **Figures 6**–**8**. The black line is predicted correctly, the red line is predicted error, and the yellow node is the core protein.

CD9 is a four-pass transmembrane protein superfamily composed of multiple homologous membrane proteins, which is widely distributed in different tissues of human body and participates in the regulation of sperm-egg binding. It plays an important role in cell membrane biology in connection with cell support, adhesion, movement, proliferation, fusion and metastasis of tumor cells. This paper uses the CD9 single-core network dataset, where a protein interacts radially with other proteins (Yang et al., 2006). The result indicates that all 16 PPIs could be identified by our method. The accuracy of this method is 18.75% higher than that of Shen's work (Juwen et al., 2007).

The Ras-Raf-Mek-Erk-Elk-Srf pathway is a widely activated mitogen-activated protein kinase signaling pathway that is complex, highly conserved and widely found in eukaryotic cells. It can transmit extracellular signals into the nucleus, causing changes in the expression profile of specific proteins in the cells, which in turn affects cell fate, and is closely related to the development of tumors (Davis, 2010). Ras, Raf, Mek, Erk, Elk, and Srf act as core proteins that determine signal transduction. Our method has a prediction accuracy of 95.96%, which is better than 85.19% of Shen's work (Juwen et al., 2007).

The Wnt signaling pathway is a group of multiple downstream channel signaling pathways that are excited by the binding of the ligand protein Wnt and membrane protein receptors. In biology, most PPIs network is the cross-connection network. While Wnt-related pathways are essential for signal transduction, the use of scientific computing methods to predict Wnt-related network has important practical significance (Stelzl et al., 2005). The accuracy of Shen's work is 96.04% in the network, our method is 100% which is best.

### Evaluating the Performance of FCTP-WSRC by PIE Software

PIE (Protein Interaction information Extraction) the search is a web service to extract PPI-relevant articles from MEDLINE (Sun et al., 2012), which can be used *via* a web application at http://www.ncbi.nlm.nih.gov/IRET/PIE/. It implement a competition-winning approach utilizing word and syntactic analyses by machine learning techniques. For easy user access, PIE the search provides a PubMed-like search environment, but the output is the list of articles prioritized by PPI confidence scores. PPI score is a relative value between 1.0 (highly likely) and -1.0 (highly unlikely) among retrieved articles. From **Table 7**, we can see that only CD9-CD59 is negative 0.0798, which is very close to zero obtained by the web tool PIE. That is to see, PPI-relevant articles extracted by the PIE cannot predict the relationship between CD9 and CD59. This also shows that our method can be used to predict potential PPI.

**Table 7:** Protein-protein interaction information obtained by a web tool PIE.

Protein-protein interaction	PMID	PPI score
CD9-CD19	9804823	0.7703
CD9-CD9 partner	16690612	0.9999
CD9-Integrin alpha 3	7790364	0.9999
CD9-Protein Kinase C alpha	11325968	0.7479
CD9-CD81 Partner 3	16690612	0.9999
CD9-CD53	23500527	0.818
CD9-CD81 antigen	16690612	0.9999
CD9-KIT	12036870	0.7073
CD9-Tetraspanin 4	27993971	0.9502
CD9-ADAM2	10518536	0.557
CD9-CD59	15625824	-0.0798
CD9-CD36	17684062	0.6525
CD9-Integrin alpha 5	10811835	0.8497
CD9-CD63 antigen	19640571	0.7556
CD9-DTS	8367482	0.1173
CD9-Collagen binding protein 2	9931299	0.5501

### Conclusion

The problem of predicting PPIs has been tackled extensively. Given the fact that computational tools for predicting PPIs have been used over years, only a few of them are able to predict easily, quickly, and accurately. Above all, we have explored a novel computational tool called FCTP-WSRC to predict PPIs efficiently. We characterize a fixed-length feature vector of protein sequence using descriptors F-vector, composition (C), and transition (T).

Our numerical results demonstrate that the WSRC classifier model is feasible to perform PPIs detection. We see that FCTP-WSRC perform significantly well when it comes to distinguish positive samples and negative samples of protein pairs. That is to say, these results support the notion that our FCTP-WSRC model is a highly effective proteomics research support tool. In the future, we will extend our approach to more significant PPI networks with unknown biological functions.

Code is programmed by MATLAB, which can be downloaded from https://github.com/wowkiekong/PPI-prediction. User-friendly and publicly accessible web-servers represent the future direction for developing practically more useful computational tools and enhancing their impact (Chou, 2017). Our future efforts will be to establish a web-server for the prediction method reported in this paper. 

## Data Availability Statement

All datasets generated for this study are included in the article/**Supplementary Material**.

## Author Contributions

MK, YZ, and DX contributed conception and design of the study. YZ and WC performed the data processing. MK and DX constructed the protein–protein interactions prediction model. MK wrote the first draft of the manuscript. YZ, WC, DX, and MD wrote sections of the manuscript. All authors contributed to manuscript revision, read, and approved the submitted version.

## Conflict of Interest

The authors declare that the research was conducted in the absence of any commercial or financial relationships that could be construed as a potential conflict of interest.
